# Supplementary material for: Realization of an inherent time crystal in a dissipative many-body system
Source: Nat Commun. 2023 Oct 3;14:6161. doi: 10.1038/s41467-023-41905-3 (PMC10547780; doi:10.1038/s41467-023-41905-3)
Supplement: Supplementary file 1 — Supplementary Information [file 41467_2023_41905_MOESM1_ESM.pdf]

Supplemental Information: Realization of an inherent time crystal in a dissipative many-body system

Yu-Hui Chen and Xiangdong Zhang

Key Laboratory of advanced optoelectronic quantum architecture and measurements of Ministry of Education,
Beijing Key Laboratory of Nanophotonics & Ultrafine Optoelectronic Systems,
School of Physics, Beijing Institute of Technology, 100081, Beijing, China

CONTENTS:

Supplementary Note 1. System Hamiltonian and mean-field approximation

Supplementary Note 2. Many-body interactions and time crystalline order

Supplementary Note 3. Feedback loop of the four-level system

Supplementary Note 4. Dipole-dipole interactions of erbium ions

Supplementary Note 5. Long time behaviours

Supplementary Note 6. Phase diagram

Supplementary Note 7. Phase transitions at different laser frequencies

Supplementary Note 8. Intrinsic optical instability without time crystalline order

Supplementary Note 9. Data analysis using cross-correlation function

Supplementary Note 10. Periodicities at different delays

Supplementary Note 11. Phase discontinuities in theoretical model

Supplementary Note 12. Differences between self pulsing and time crystal

Supplementary Note 1. System Hamiltonian and mean-field approximation

We consider a collection of four-level atoms whose energy structure consists of two pairs of Kramers doublets separated by an optical transition. The Hamiltonian of an ensemble of CW-pumped four-level atoms is given by

$$\begin{aligned} H_{\text{sys}} = \sum_i \Big(\sum_{g,e} \delta_{g,i}|g\rangle\langle g|_i + \Delta_{e,i}|e\rangle\langle e|_i + \Omega_{ge}(\mathbf{r}_i)|g\rangle\langle e|_i + \Omega_{ge}(\mathbf{r}_i)|e\rangle\langle g|_i \Big) \\ + \sum_{i,j} \frac{J_{ij}}{2\left|\mathbf{r}_{ij}\right|^3}[\mathbf{S}_i\cdot\mathbf{S}_j - 3(\mathbf{S}_i\cdot\widehat{\mathbf{r_{ij}}})(\mathbf{S}_j\cdot\widehat{\mathbf{r_{ij}}})], \end{aligned} \tag{S.1}$$

where $|g\rangle_i$ and $|e\rangle_i$ indicate the optical ground and excited states of the ith atom, $g = 1, 2$ and $e = 1, 2$ means that there are a pair of Kramers doublets in the ground and excited states, $\delta_{g,i}$ is the ground-state detuning, $\Delta_{e,i}$ is the excited-state detuning, $\Omega_{ge}(\mathbf{r}_i)$ is the optical Rabi frequency of the ith atom, J_{ij} is the strength of the magnetic dipole interactions, $\mathbf{r}_{i,j}$ is the vector connecting two spins $\mathbf{S}_i$ and $\mathbf{S}_j$, and $\widehat{\mathbf{r}_{ij}}$ is the corresponding unit vector. The system Hamiltonian H_{sys} is the sum of the Hamiltonian of individual ion H_i, which means that $H_{\text{sys}} = \sum_i H_i$ with

$$\begin{aligned} H_i = \sum_{g,e} \delta_{g,i}|g\rangle\langle g|_i + \Delta_{e,i}|e\rangle\langle e|_i + \Omega_{ge}(\mathbf{r}_i)|g\rangle\langle e|_i + \Omega_{ge}(\mathbf{r}_i)|e\rangle\langle g|_i \\ + \sum_{j\neq i} \frac{J_{ij}}{2\left|\mathbf{r}_{ij}\right|^3}[\mathbf{S}_i\cdot\mathbf{S}_j - 3(\mathbf{S}_i\cdot\widehat{\mathbf{r_{ij}}})(\mathbf{S}_j\cdot\widehat{\mathbf{r_{ij}}})]. \end{aligned} \tag{S.2}$$

In principle, the parameters in H_i, such as $\delta_{g,i}$ and $J_{i,j}$, varies from atom to atom. It is impractical to calculate in a full quantum way the response of such a system. Here we consider that the atoms are homogeneous, i.e., $\delta_{g,i} \equiv \delta_g$, $\Delta_{e,i} \equiv \Delta_e$, $\Omega_{ge}(\mathbf{r}_i) \equiv \Omega_{ge}$. Then the Hamiltonian H_i becomes

$$H_i = \sum_{g,e} \Big(\delta_g|g\rangle\langle g| + \Delta_e|e\rangle\langle e| + \Omega_{ge}|g\rangle\langle e| + \Omega_{ge}|e\rangle\langle g|\Big) + \sum_{j\neq i}\frac{V_{i,j}}{2}, \tag{S.3}$$

where the interaction term is rewritten as $V_{i,j}$. The dipole-dipole interactions $V_{i,j}$ in our theoritical model depends on the relative position $\mathbf{r}_{ij}$ of the ions and the relative orientation of the dipoles. This interaction presents two main features: a long-range character through the $1/r^3$ decay (instead of a short-range $1/r^6$ decay) and an anisotropic nature in space. The total interaction on the ith ion is the sum of the contribution of all the jth ions, i.e., $V_i = \sum_j V_{i,j}$. An intuitive way to see the long-range behaviour of such an interaction is to use an integral to replace the sum, i.e., $V_i = \int V(\mathbf{r}_{i,j})d\mathbf{r}_j$. As the number of interacting ions with similar interaction strength $V(\mathbf{r}_{i,j}) = V(r) \propto 1/r^3$ grows with r^2. Then the total interaction strength on the ith ion V_i can be estimated by an integral of the form of $\int r^2 \cdot 1/r^3 dr$. This equation suggests that the integral grows with increasing r and even become divergent when the crystal size $r \to \infty$. Therefore, the dipole-dipole interaction in our sample is long range. To precisely calculate V_i, a more dedicate model involving the microscopic information of the doped erbium ions is needed. Here, we apply the mean-field approximation [1, 2] to study the influence of the term V_i. The mean-field theory means that the density matrix of the whole system can be factorized $\rho_{\text{sys}} = \bigotimes_k \rho_k$, and the reduced density matrix of the ith atom is $\rho_i = \text{Tr}_{\neq i}(\rho_{\text{sys}})$.

The equation of motion for the system is $\dot{\rho_{\text{sys}}} = -i[\rho_{\text{sys}}, H_{\text{sys}}]$. Then the dynamic equation of the reduced ρ_i is

$$\begin{aligned} \dot{\rho}_i &= \frac{d}{dt}\Big[\text{Tr}_{\neq i}(\rho_{\text{sys}})\Big] \\ &= \text{Tr}_{\neq i}\Big(\dot{\rho_{\text{sys}}}\Big) \\ &= -i\,\text{Tr}_{\neq i}\Big(\rho_{\text{sys}}H_{\text{sys}} - H_{\text{sys}}\rho_{\text{sys}}\Big) \\ &= -i\,\text{Tr}_{\neq i}\Big(\rho_{\text{sys}}\sum_m H_m - \sum_m H_m\rho_{\text{sys}}\Big). \end{aligned} \tag{S.4}$$

Note that H_m is given by Eq. (S.3)

$$\begin{aligned} H_m &= \sum_{g,e} \Big(\delta_g|g\rangle\langle g| + \Delta_e|e\rangle\langle e| + \Omega_{ge}|g\rangle\langle e| + \Omega_{ge}|e\rangle\langle g| + \sum_{n\neq m}\frac{V_{m,n}}{2} \\ &\equiv h_m + \sum_{n\neq m}\frac{V_{m,n}}{2}, \end{aligned} \tag{S.5}$$

where h_m contains the detuning terms and the optical driving terms, and $V_{m,n}$ stands for the many-body interactions. For the first term h_m in Eq. (S.5), we then obtain

$$\begin{aligned}
\mathrm{Tr}_{\neq i}\Big(\rho_{\rm sys}\sum_m h_m - \sum_m h_m\rho_{\rm sys}\Big) &= \mathrm{Tr}_{\neq i}\Big(\bigotimes \rho_k \sum_m h_m - \sum_m h_m \bigotimes \rho_k\Big) \\
&= \mathrm{Tr}_{\neq i}\Big\{\sum_m \Big[(\rho_m h_m - h_m\rho_m)\bigotimes_{k\neq m}\rho_k\Big]\Big\} \\
&= \mathrm{Tr}_{\neq i}\Big[(\rho_i h_i - h_i\rho_i)\bigotimes_{k\neq i}\rho_k\Big] + \mathrm{Tr}_{\neq i}\Big[\sum_{m\neq i}(\rho_m h_m - h_m\rho_m)\bigotimes_{k\neq m}\rho_k\Big] \\
&= (\rho_i h_i - h_i\rho_i)\mathrm{Tr}_{\neq i}\Big(\bigotimes_{l\neq i}\rho_l\Big) - 0 \\
&= (\rho_i h_i - h_i\rho_i),
\end{aligned} \tag{S.6}$$

where we have used $\mathrm{Tr}_{\neq i}\big(\bigotimes_{k\neq i}\rho_k\big) = 1$ and $\mathrm{Tr}(\rho_m h_m - h_m\rho_m) = 0$. This equation means a well-known result that $\dot{\rho}_i = -i[\rho_i, h_i]$ when there is no cross talk $V_{m,n}$ between different atoms.

For the second term $V_{m,n}$ in Eq. (S.5), following a similar procedure, we obtain

$$\begin{aligned}
\mathrm{Tr}_{\neq i}\Big(\rho_{\rm sys}\sum_{m,n} V_{m,n} - \sum_{m,n} V_{m,n}\rho_{\rm sys}\Big) =& \mathrm{Tr}_{\neq i}\Big(\bigotimes \rho_k \sum_{m,n} V_{m,n} - \sum_{m,n} V_{m,n} \bigotimes \rho_k\Big) \\
=& \mathrm{Tr}_{\neq i}\Big\{\sum_{m,n}\Big[(\rho_m\otimes\rho_n\cdot V_{m,n} - V_{m,n}\cdot\rho_m\otimes\rho_n)\bigotimes_{k\neq m,n}\rho_k\Big]\Big\} \\
=& \mathrm{Tr}_{\neq i}\Big[(\sum_n \rho_i\otimes\rho_n\cdot V_{i,n} - \sum_n V_{i,n}\cdot\rho_i\otimes\rho_n)\bigotimes_{k\neq i,n}\rho_k\Big] + \\
& \mathrm{Tr}_{\neq i}\Big[(\sum_m \rho_m\otimes\rho_i\cdot V_{m,i} - \sum_m V_{m,i}\cdot\rho_m\otimes\rho_i)\bigotimes_{k\neq i,m}\rho_k\Big] + \\
& \mathrm{Tr}_{\neq i}\Big[(\sum_{m\neq i,n\neq i} \rho_m\otimes\rho_n\cdot V_{m,n} - \sum_{m\neq i,n\neq i} V_{m,n}\cdot\rho_m\otimes\rho_n)\bigotimes_{k\neq m,n,i}\rho_k\Big]\cdot\rho_i.
\end{aligned} \tag{S.7}$$

For the first term in the above equation, we have

$$\begin{aligned}
\mathrm{Tr}_{\neq i}\Big[(\sum_n \rho_i\otimes\rho_n\cdot V_{i,n} - \sum_n V_{i,n}\cdot\rho_i\otimes\rho_n)\bigotimes_{k\neq i,n}\rho_k\Big] =& \mathrm{Tr}_n\Big[(\sum_n \rho_i\otimes\rho_n\cdot V_{i,n} - \sum_n V_{i,n}\cdot\rho_i\otimes\rho_n)\Big]\cdot\mathrm{Tr}_{\neq i,n}\Big(\bigotimes_{k\neq i,n}\rho_k\Big) \\
=& \mathrm{Tr}_n\Big[(\sum_n \rho_i\otimes\rho_n\cdot V_{i,n} - \sum_n V_{i,n}\cdot\rho_i\otimes\rho_n)\Big] \\
=& \rho_i\otimes\mathrm{Tr}_n\Big(\sum_n \rho_n\cdot V_{i,n}\Big) - \mathrm{Tr}_n\Big(\sum_n V_{i,n}\cdot\rho_n\Big)\otimes\rho_i.
\end{aligned} \tag{S.8}$$

Similarly, the second term in Eq. (S.7) becomes

$$\mathrm{Tr}_{\neq i}\Big[(\sum_m \rho_m\otimes\rho_i\cdot V_{m,i} - \sum_m V_{m,i}\cdot\rho_m\otimes\rho_i)\bigotimes_{k\neq i,m}\rho_k\Big] = \rho_i\otimes\mathrm{Tr}_m\Big(\sum_m \rho_m\cdot V_{m,i}\Big) - \mathrm{Tr}_m\Big(\sum_m V_{m,i}\cdot\rho_m\Big)\otimes\rho_i. \tag{S.9}$$

Using again that $\mathrm{Tr}(AB - BA) = 0$, we obtain for the third term in Eq. (S.7) that

$$\mathrm{Tr}_{\neq i}\Big[(\sum_{m\neq i,n\neq i} \rho_m\otimes\rho_n\cdot V_{m,n} - \sum_{m\neq i,n\neq i} V_{m,n}\cdot\rho_m\otimes\rho_n)\bigotimes_{k\neq m,n}\rho_k\Big] = 0. \tag{S.10}$$

Substituting Eq. (S.8), (S.9) and (S.10) into Eq. (S.7), we have

$$\frac{1}{2}\mathrm{Tr}_{\neq i}\Big(\rho_{\rm sys}\sum_{m,n} V_{m,n} - \sum_{m,n} V_{m,n}\rho_{\rm sys}\Big) = \rho_i\otimes\mathrm{Tr}_n\Big(\sum_n \rho_n\cdot V_{i,n}\Big) - \mathrm{Tr}_n\Big(\sum_n V_{i,n}\cdot\rho_n\Big)\otimes\rho_i. \tag{S.11}$$

By expanding $V_{i,n}$ in the basis of $|g\rangle$ and $|e\rangle$, we can rewrite it as

$$\begin{aligned}
V_{i,n} = \sum_{g_i,g_n,e_i,e_n} & \langle g_i g_n|V_{i,n}|g_i g_n\rangle\cdot|g_i g_n\rangle\langle g_i g_n| + \langle g_i e_n|V_{i,n}|g_i e_n\rangle\cdot|g_i e_n\rangle\langle g_i e_n| \\
& + \langle e_i g_n|V_{i,n}|e_i g_n\rangle\cdot|e_i g_n\rangle\langle e_i g_n| + \langle e_i e_n|V_{i,n}|e_i e_n\rangle\cdot|e_i e_n\rangle\langle e_i e_n|.
\end{aligned} \tag{S.12}$$

In the above expansion, those terms as $|g_i g_n\rangle\langle g_i e_n|$ are dropped under the rotating wave approximation, and the energy transfer terms as $|g_i e_n\rangle\langle e_i g_n|$ are assumed to be negligible. Substituting Eq. (S.12) into the tracing $\mathrm{Tr}_n\Big(\sum_n \rho_n \cdot V_{i,n}\Big)$ in Eq. (S.11) gives

$$\begin{aligned}\mathrm{Tr}_n\Big(\sum_n \rho_n \cdot V_{i,n}\Big) = &\sum_{g_i,g_n,e_i,e_n} \langle g_i g_n|V_{i,n}|g_i g_n\rangle \cdot \rho_{gg,n} \cdot |g\rangle\langle g|_i + \langle g_i e_n|V_{i,n}|g_i e_n\rangle \cdot \rho_{ee,n} \cdot |g\rangle\langle g|_i \\ &\quad + \langle e_i g_n|V_{i,n}|e_i g_n\rangle \cdot \rho_{gg,n} \cdot |e\rangle\langle e|_i + \langle e_i e_n|V_{i,n}|e_i e_n\rangle \cdot \rho_{ee,n} \cdot |e\rangle\langle e|_i \\ \overset{\text{def}}{=} &\sum_{g_i,g_n,e_i,e_n} V_a \cdot \rho_{gg,n}|g\rangle\langle g|_i + V_b \cdot \rho_{ee,n}|g\rangle\langle g|_i + V_c \cdot \rho_{gg,n}|e\rangle\langle e|_i + V_d \cdot \rho_{ee,n}|e\rangle\langle e|_i,\end{aligned} \tag{S.13}$$

where the coefficients $V_a = \langle g_i g_n|V_{i,n}|g_i g_n\rangle$ and so do V_b, V_c and V_d. It is clear that the effect of $V_{m,n}$ is to shift the energy of the states $|g\rangle$ and $|e\rangle$. Generally, the energy shifts for the ground and excited states are not the same. In optical experiments where a laser field drives the transitions between the states of $|g\rangle$ and $|e\rangle$, what matters is the frequency differences between them. Therefore, the induced frequency difference χ of the optical ground and excited states of the ith ion becomes

$$\begin{aligned}\chi_n &= \sum_{g_n,e_n} V_c\,\rho_{gg,n} + V_d\,\rho_{ee,n} - V_a\,\rho_{gg,n} - V_b\,\rho_{ee,n} \\ &= \sum_{g_n,e_n} \frac{V_c + V_d - V_a - V_b}{2}(\rho_{gg,n} + \rho_{ee,n}) + \frac{V_a + V_d - V_b - V_c}{2}(\rho_{ee,n} - \rho_{gg,n}).\end{aligned} \tag{S.14}$$

Noting that $\sum_{g,e} \rho_{gg,n} + \rho_{ee,n} = 1$, we can neglect the first term in the above equation as it simply implies a constant frequency shift. Then we obtain

$$\chi_n = \sum_{g_n,e_n} \eta_n(\rho_{ee,n} - \rho_{gg,n}), \tag{S.15}$$

where $\eta_n = (V_a + V_d - V_b - V_c)/2$. It is now clear that the effect of ion-ion interactions $V_{m,n}$ is to introduce an excitation-dependant frequency shift to the ith ion. With Eq. (S.3) and (S.15), we can write the Hamiltonian of the ith erbium ion as

$$H_i = h_i + \sum_n V_{i,n} = \sum_{g_i,e_i} \delta_g|g\rangle\langle g| + \Delta_e|e\rangle\langle e| + \Omega_{ge}|g\rangle\langle e| + \Omega_{ge}|e\rangle\langle g|_i + \sum_n \sum_{g_n,e_n,e_i} \eta_n(\rho_{ee,n} - \rho_{gg,n})|e\rangle\langle e|_i. \tag{S.16}$$

Hamiltonians of a similar form as Eq. (S.16) are widely used to analyze phase transitions in electron spins [3], Rydberg atoms [2, 4], and rare-earth ions[5, 6]. For a uniform version of Eq. (S.16), that is, the density matrix is the same for all ions ($\rho_{ee,i} = \rho_{ee,n} \equiv \rho_{ee}$ and $\rho_{gg,i} = \rho_{gg,n} \equiv \rho_{gg}$), we can obtain

$$H_i = \sum_{g,e} \Big(\delta_g|g\rangle\langle g| + \Delta_e|e\rangle\langle e| + \Omega_{ge}|g\rangle\langle e| + \Omega_{ge}|e\rangle\langle g| + \Delta_s \sum_{g',e'}(\rho_{e'e'} - \rho_{g'g'})|e\rangle\langle e|\Big), \tag{S.17}$$

where $\Delta_s = \sum_n \eta_n$ is the excitation-induced frequency shift. This kind of frequency shift is often considered to be a decoherence source [7] and recently be employed to demonstrate quantum gate operations [8] and conditional quantum phase shift [9]. Moreover, such a nonlinear effect is also the origin of intrinsic optical intrinsic optical instabilities [6, 10]. Rewriting Eq. (S.17) explicitly for a four-level system, we have

$$\begin{aligned}H_i =&\delta_2\sigma_{22} + \delta_3\sigma_{33} + \delta_4\sigma_{44} \\ &+\Omega_{13}\sigma_{13} + \Omega_{14}\sigma_{14} + \Omega_{23}\sigma_{23} + \Omega_{24}\sigma_{24} \\ &+\Delta_s(\rho_{44} + \rho_{33} - \rho_{22} - \rho_{11})\sigma_{33} + \Delta_s(\rho_{44} + \rho_{33} - \rho_{22} - \rho_{11})\sigma_{44}.\end{aligned} \tag{S.18}$$

With this Hamiltonian, we can now compute the Lindblad equation $\dot{\rho} = L\rho$ by using the Runge-Kutta method (Notre 2 or more detailed in our previous work[6]). In our experiment, the measured light intensity $I(t)$ is related to $\rho(t)$ through the macroscopic polarization $P(z,t)$. We consider a laser field $\mathcal{E}(z,t) = E(z,t)\exp(-i\omega t + ikz + \phi)$ passing through our sample along the z direction from $z = 0$ to $z = L$, where L is the length of our sample. The intensity $I(t)$ is proportional to $|E(z = L, t)|^2$. Applying the slowly-varying-amplitude approximation to $E(z,t)$, we have

$$\frac{\partial}{\partial z}E(z,t) = \frac{k}{2\epsilon} \cdot \mathrm{Im}P(z,t), \tag{S.19}$$

where k is the wave vector, ϵ is the permittivity constant, and $\mathrm{Im}P(z,t)$ is the imaginary part of the polarization $P(z,t)$. Given the density of erbium ions n_{er}, the polarization $P(z,t)$ is related to the above-calculated $\rho(t)$ by

$$P(z,t) = n_{\mathrm{er}}[d_{12} \cdot \rho_{12}(z,t) + d_{13} \cdot \rho_{13}(z,t) + d_{23} \cdot \rho_{23}(z,t) + d_{24} \cdot \rho_{24}(z,t)]. \tag{S.20}$$

We then obtain

$$E(L,t) = \int_0^L dz \cdot \frac{n_{\rm er}k}{2\epsilon} \cdot {\rm Im}[d_{12} \cdot \rho_{12}(z,t) + d_{13} \cdot \rho_{13}(z,t) + d_{23} \cdot \rho_{23}(z,t) + d_{24} \cdot \rho_{24}(z,t)] \quad \text{(S.21)}$$

Since we are only interested in the temporal response of our system, Eq. (S.21) clearly shows that an oscillating $\rho(t)$ can result in a oscillating $E(L,t)$ and $I(t)$.

Note that the Hamiltonian Eq. (S.18) is based on a homogeneous model. However, erbium ions doped in crystals are subject to inhomogeneous broadening. This means that the frequencies of their optical transitions and spin transitions can vary from ion to ion within the crystal. The inhomogeneous broadening of optical transition is approximately 1 GHz and that of spin transitions is on the order of 10 MHz. In our system, the absolute optical transition frequency is not important as it can be compensated by tuning the laser frequency. What really matters is the detuning terms such as δ_2, δ_3 and δ_4. Applying a laser of mw power to drive our erbium ensemble results in approximately 10% of the ion in the inhomogeneous line are excited. This implies that the δ_2, δ_3 and δ_4 corresponding to the excited ions vary within a range of ~1 MHz. We thus assuming that the atomic detunings are the same in our theoretical model. Note that in our experiments, some ions with different frequency detunings can also be excited. However, they are likely not in a dynamically stable phase and can just impose a cw background in our time-crystal detection.

Supplementary Note 2. Many body interactions and time crystalline order

I. Many-body interations

The Hamiltonian Eq. S.18 can be represented as a matrix and consists of three parts:

$$H = H_0 + H_r + H_m \quad \text{(S.22)}$$

where

$$H_0 = \begin{bmatrix} 0 & 0 & 0 & 0 \\ 0 & \delta_2 & 0 & 0 \\ 0 & 0 & \delta_3 & 0 \\ 0 & 0 & 0 & \delta_4 \end{bmatrix}, \quad \text{(S.23)}$$

represents the Hamiltonian of free ions,

$$H_r = \begin{bmatrix} 0 & 0 & \Omega t_1 & \Omega t_2 \\ 0 & 0 & \Omega t_2 & \Omega t_1 \\ \Omega t_1 & \Omega t_2 & 0 & 0 \\ \Omega t_2 & \Omega t_1 & 0 & 0 \end{bmatrix}, \quad \text{(S.24)}$$

is the Rabi oscillation term governed by the optical driving, and

$$H_m = \begin{bmatrix} 0 & 0 & 0 & 0 \\ 0 & 0 & 0 & 0 \\ 0 & 0 & \Delta_s(\rho_{44}+\rho_{33}-\rho_{22}-\rho_{11}) & 0 \\ 0 & 0 & 0 & \Delta_s(\rho_{44}+\rho_{33}-\rho_{22}-\rho_{11}) \end{bmatrix}, \quad \text{(S.25)}$$

represents the many-body interactions.

The expression of H_m indicates that when the input laser is weak (or absent), most erbium ions remain in their ground states. In this case, H_m acts as a constant frequency shift on the states $|3\rangle$ and $|4\rangle$. However, as more ions are excited and the values of ρ_{33} and ρ_{44} become significant, the frequencies of the targeted ions are altered. This kind of many-body interactions serves as the underlying mechanism for various quantum phenomena, including quantum gate operations, intrinsic optical bistability, and intrinsic optical instability [6–8].

With the above Hamiltonian and the density matrix of an erbium ion

$$\rho = \begin{bmatrix} \rho_{11} & \rho_{12} & \rho_{13} & \rho_{14} \\ \rho_{21} & \rho_{22} & \rho_{23} & \rho_{24} \\ \rho_{31} & \rho_{32} & \rho_{33} & \rho_{34} \\ \rho_{41} & \rho_{42} & \rho_{43} & \rho_{44} \end{bmatrix}, \quad \text{(S.26)}$$

one can compute the time evolution of $\rho(t)$ through the Lindblad equation

$$\frac{d}{dt}\rho = -i[H,\rho] + Loss, \quad \text{(S.27)}$$

The *Loss* term accounts for all the decay and dephasing processes of the erbium ions. It encompasses various rates, including the spin relaxation rate γ_{12} between $|2\rangle$ and $|1\rangle$, the spin relaxation rate γ_{34} between $|4\rangle$ and $|3\rangle$, the optical spontaneous emission rate γ_{31} (γ_{32}) from $|3\rangle$ to $|1\rangle$ ($|2\rangle$), the optical spontaneous emission rate γ_{41} (γ_{42}) from $|4\rangle$ to $|1\rangle$ ($|2\rangle$), the spin dephasing rates $\gamma_{22}, \gamma_{33}, \gamma_{44}$ of $|2\rangle$, $|3\rangle$ and $|4\rangle$, respectively [6]. These loss terms in the Lindblad form can be expressed as

$$L(\sigma_{jk}) = \frac{\gamma_{jk}}{2}(n_{jk}+1)\cdot(2\sigma_{jk}\rho\sigma_{kj} - \sigma_{kj}\sigma_{jk}\rho - \rho\sigma_{jk}\sigma_{kj}) + \frac{\gamma_{jk}}{2}n_{jk}\cdot(2\sigma_{kj}\rho\sigma_{jk} - \sigma_{jk}\sigma_{kj}\rho - \rho\sigma_{kj}\sigma_{jk}), \tag{S.28}$$

where n_{jk} is the average photon number of the thermal bath at the frequency between level j and k.

The parameters used in obtaining the results in Fig. 1 of the manucript are listed below. The detunings are $\delta_2 = 0.05$ MHz, $\delta_3 = -0.35$ MHz, and $\delta_4 = 0.4$ MHz. The induced-frequency shift is $\Delta_s = 12$ MHz. The dephasing rates are $\gamma_{22} = \gamma_{33} = \gamma_{44} = 1$ kHz. The optical coupling strengths of $|1\rangle$ to $|3\rangle$ and that of $|1\rangle$ to $|4\rangle$ are Ωt_1 and Ωt_2, respectively, where $\Omega = 0.26$ MHz, $t_1 = 1.87$ and $t_2 = 1.2$. Similarly, the optical coupling strengths of $|2\rangle$ to $|3\rangle$ and that of $|2\rangle$ to $|4\rangle$ are Ωt_2 and Ωt_1, respectively. The optical excited states have a lifetime of $1/\gamma = 11$ ms. The spin relaxation time for $|2\rangle$ to $|1\rangle$ and $|4\rangle$ to $|3\rangle$ are chosen to be 2 seconds. The longest time scale of the erbium dynamics in our simulations is 0.17 s, equivalent to 15 times the optical excited state lifetime of erbium ions. The oscillation depicted in Fig. 1 remains clear and unattenuated throughout the entire simulation period.

The H_m in Eq. (S.18) represents the many-body interactions, which play a crucial role in generating the time crystalline order in our system. Without these interactions, self-sustained oscillations would be absent. To illustrate this effect, we computed the time evolution of $\rho(t)$ for various values of Δ_s. Figure S1 shows the calculated $\rho_{33}(t)$ for different Δ_s. When there are no ion-ion interactions ($\Delta_s = 0$ MHz), Rabi oscillations on the order of MHz can be observed shortly after the driving field is switched on (inset of Fig. S1(a)). However, the system quickly stabilizes at approximately 5 ms, indicating the absence of time crystalline order. If we include ion-ion interactions in the model with a small magnitude ($\Delta_s = 4$ MHz), the $\rho_{33}(t)$ remains stationary in the long time limit. When Δ_s is increased to 8 MHz, the $\rho_{33}(t)$ in the long time limit becomes dynamically unstable, indicating the spontaneous breaking of the continuous time translation symmetry. Further increasing Δ_s to 10 MHz leads to an even more pronounced instability of $\rho_{33}(t)$, further confirming the effect of the many-body interactions. The many-body interactions between erbium ions act as nonlinear intrinsic feedback, amplifying small perturbations and driving the erbium ensemble into a new stable dissipative order. Without these many-body interactions, no temporal instability or periodicity can be generated in our system. These results are consistent with the results reported in our previous publications [6].

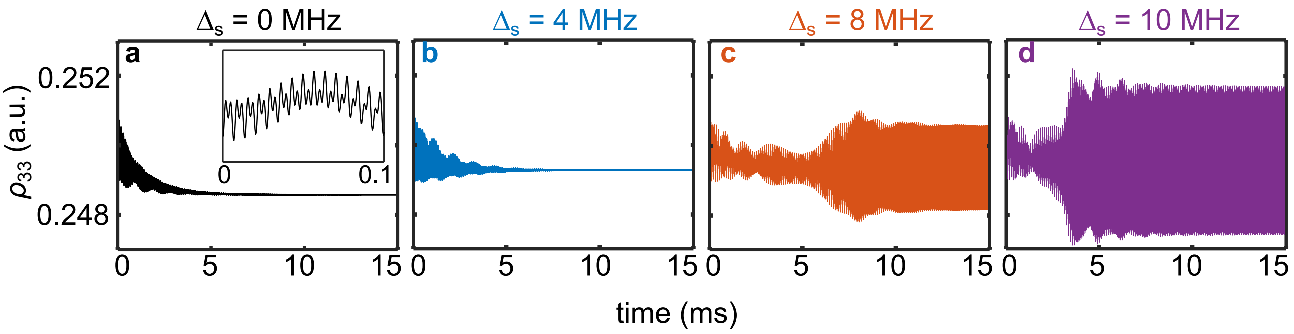

FIG. S1. **Population $\rho_{33}(t)$ for different many-body interactions Δ_s.** (**a**)-(**d**), calculated population $\rho_{33}(t)$ for different Δ_s as noted. When the time translation invariance of $\rho_{33}(t)$ is broken for $\Delta_s = 8$ MHz and 10 MHz, Rabi oscillations can also be observed throughout the entire time range.

II. Time crystalline order

When the time translation invariance of $\rho(t)$ is broken in the long time limit due to the inclusion of many-body interactions, Rabi oscillations induced by the optical driving can still be observed throughout the entire time range. This suggests that the system continues to undergo coherent population oscillations between the optical ground and excited states. More importantly, under appropriate parameters, the unstable $\rho(t)$ exhibits self-sustained oscillations at frequencies different from the Rabi oscillation frequencies. These regular oscillations indicate the formation of temporal order. For example, in Fig. S2(a), the Fourier spectrum of the calculated $\rho(t)$ (Fig. 1C in the main text) shows a peak at 46.4 kHz, confirming the presence of temporal order.

Within the large parameter space of the four-level systems, it is found that the characteristic frequency of the inherent time crystal is sensitive to the optical transition overlaps. Specifically, we set the optical coupling strengths of $|1\rangle$ to $|3\rangle$ and that of $|1\rangle$ to $|4\rangle$ to be Ωt_1 and Ωt_2, respectively, as shown in Fig.S2b. Here Ω depends on the pump laser, and t_1 and t_2 are coupling coefficients. Similarly, the optical coupling strengths of $|2\rangle$ to $|3\rangle$ and that of $|2\rangle$ to $|4\rangle$ are Ωt_2 and Ωt_1, respectively. After obtaining the time response of $\rho(t)$, we then carry out a Fourier transform to $\rho(t)$ in the long time scale. The time-crystal

frequency as a function of the ratio of t_1/t_2 is plotted in Fig. S2c. For a range of t_1/t_2 varying from 1.08 to 1.20, the crystal frequency changes from 8.3 to 20.7 kHz. The result suggests a strong dependence of the crystal frequency on the ratio of t_1/t_2.

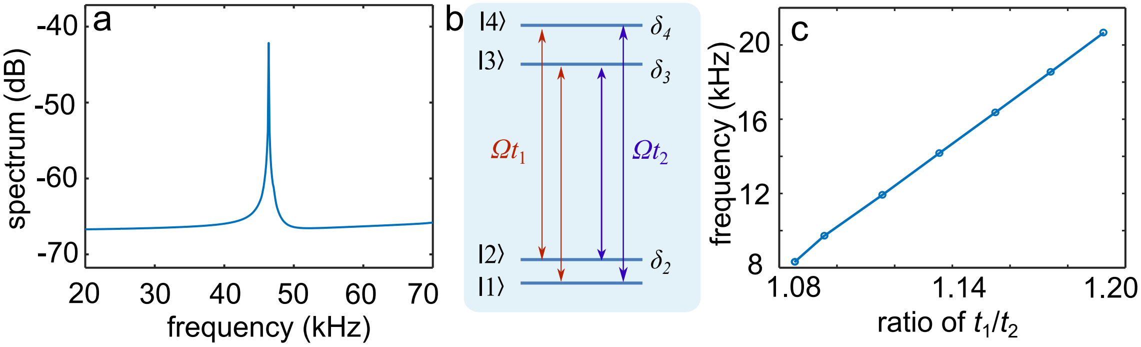

FIG. S2. **Time-crystal frequency as a function of** t_1/t_2**.** (**a**) The Fourier spectrum of $\rho_{11}(t)$ in Fig. 1C in the main text. (**b**) The four-level energy structure of an erbium ion. The transition strength between $|1\rangle$ and $|3\rangle$ and that between $|2\rangle$ and $|4\rangle$ are Ωt_1. The transition strength between $|1\rangle$ and $|4\rangle$ and between $|2\rangle$ and $|3\rangle$ are Ωt_2. (**c**) The time-crystal frequency as a function of the ratio t_1/t_2.

The physics of such dependence can be understood from Fig. 1D and 1E in the main text. As discussed, it is essential to have enough complexity in the energy structure to enable competition between different optical transitions. Such competition is necessary to bring an ensemble of atoms into a dynamically unstable phase. For pure two-level systems, the lack of complexity prevents such competition between different optical transitions and thus rules out a temporal-order phase[6, 11]. For four-level systems, the ratio t_1/t_2 indicates the overlaps between the different transitions. Therefore, it is an important index of the competing processes and can significantly affect the time crystal frequency.

In contrast, our calculated results suggest the lifetime or the decoherence time of individual erbium ions has no significant impact on the time scale of the crystalline oscillation. The dissipation terms of erbium ions are more important in determining whether a time crystal phase can be form. While a too-low damping rate might not be enough to efficiently expel the heating due to the dynamic instability, a high rate can cause the rapid dephasing of the oscillations.

It is worth noting that the emergence of time crystalline order in our four-level system is not simply a result of combining different Rabi oscillations. As shown in Figure S1, without the presence of many-body interactions, these Rabi oscillations would only last for a short period of time and eventually fade out, indicating that the time crystalline behaviour cannot be solely explained by Rabi oscillations. Furthermore, if the time crystal were solely the effect of Rabi oscillations, we would expect the crystal frequency to be affected by both the Rabi frequency and the ratio of transitions, which contradicts our calculations and experimental results.

Supplementary Note 3. Feedback loop of the four-level system

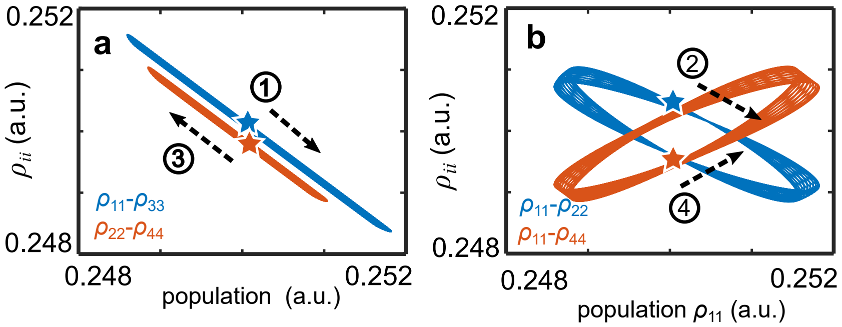

FIG. S3. **Feedback loop.** If there is a small perturbation to ρ_{11}, the system behaves as positive feedback following the steps as noted by the circled numbers. (**a**) The relationship of $\rho_{11}(t)$ and $\rho_{33}(t)$, and that of $\rho_{22}(t)$ and $\rho_{44}(t)$ in the long time limit. (**b**) The relationship of $\rho_{11}(t)$ and $\rho_{22}(t)$, and that of $\rho_{11}(t)$ and $\rho_{44}(t)$ in the long time limit.

We use the calculated results in Fig. 1 in the main text to demonstrate the feedback loop of the four-level system. Starting for the steady solution corresponding to $\dot{\rho(t)} = 0$, we assume that there is a small positive perturbation to ρ_{11}, as marked by ① in Fig. S3a. According to Fig. S3b, such an increase in ρ_{11} will lead to a decrease of ρ_{22}, as marked by ②. Then the decrease

of ρ_{22} will result in a growth of ρ_{44}, as marked by ③ in Fig. S3a. Finally, the increased ρ_{44} further enhances the increase of ρ_{11} as marked by ④ in Fig. S3b. These processes form intrinsic positive feedback inside the four-level system. The positive feedback, together with the optical transitions that compete with each other, leads the system to a dynamical instability and a self-organized periodic temporal pattern.

The limit cycles as shown in S3 is a typical sign of the breaking of time translation symmetry [12, 13]. In the atom-cavity time crystal [12], if the coupling between the cavity field and the atomic polarization is linear, the system relaxes to a stationary state at the long-time limit. The limit-cycle behaviours therein are the result of the nonlinear interaction between the cavity field and the atomic polarization. In our system, the nonlinear interactions between different electronic transitions offer the possibility of breaking time translation symmetry. The two systems share similarity in math representations, as both the cavity field and the electronic transition can be represented by an oscillator in math.

Supplementary Note 4. Dipole-dipole interactions of erbium ions

Our $Er{:}Y_2SiO_5$ crystal has a concentration of 1000 ppm, corresponding to an average distance of 4 nm between nearby erbium ions. The magnetic dipole-dipole interaction of erbium ions separated by 4 nm is $O(10\,\mathrm{MHz})$. Note that erbium ions also possess electric dipole moments. It is estimated that the electric dipole induced Stark-shift of our system is also $O(10\,\mathrm{MHz})$. Thus the contributions to the excitation-induced-frequency-shift Δ_s in Eq. (S.18) from the magnetic and the electric interactions may have a comparable magnitude[6]. However, it is not important in our case whether the Δ_s are magnetic or electric. These two types of interactions have similar mathematical forms and the same nonlinear effect on our system. Without losing generality, we here considered the many-body erbium interactions being magnetic.

Our theory predicts that a collection of four-level atoms with dipole-dipole interactions can give rise to an inherent time crystalline phase. When choosing a proper material to realize inherent time crystal, it is important to make sure the many-body interactions play a role. Therefore the ratio between the dipole-dipole interactions, optical Rabi frequency, and the dephasing rate should be high. Otherwise, the many-body interactions can not be distinguished from the background consisting of various decoherence processes. Although the erbium-erbium interactions are not as strong as that of Rydberg atoms[2], erbium-doped crystals feature extremely long optical and spin coherence times[14, 15]. Therefore the dipole-dipole interactions between nearby erbium ions are experimentally distinguishable[5], providing an experimental platform of investigating the formation for inherent time crystals.

Supplementary Note 5. Long time behaviors

Figure 2 of the main text shows the spontaneously breaking of continuous time translation symmetry and the forming of temporal order in a time scale of 20 ms. In our experiment, the oscillating $I(t)$ lasts for ever as long as the pump laser is on. This is in contrast to the continuous time crystal recently demonstrated[16], which has a lifetime limited by the atom loss. The persistence of $I(t)$ in our experiment is explicitly shown in Fig. S4, where the $I(t)$ after the laser ~100 ms after the switching of the laser is shown. As long as the pump laser is on its cw mode, such oscillations can be observed. This property is crucial as it enables us to monitor the spectra of $I(t)$ in the long-time limit (or cw mode), as shown in Fig. 3 in the main text.

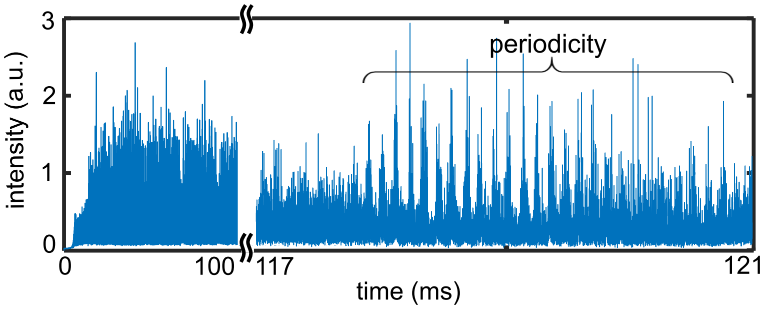

FIG. S4. **Long time behaviors of the system.** An example of $I(t)$ measured from $t = 0$ ms to $t = 121$ ms. Note that the $I(t)$ of the first 100 ms and the $I(t)$ from 117 ms to 120 ms have different scales on their x-axes.

Supplementary Note 6. Phase diagram

Figure S5 shows the phase diagram of our system. Normally the output $I(t)$ of our measurement is dynamically stable for different combination of pump power $P_{\rm in}$ and laser frequency f_l. Starting from a cw state, the output of our system under increasing $P_{\rm in}$ first becomes dynamically unstable, then temporally periodic and finally dynamically irregular again.

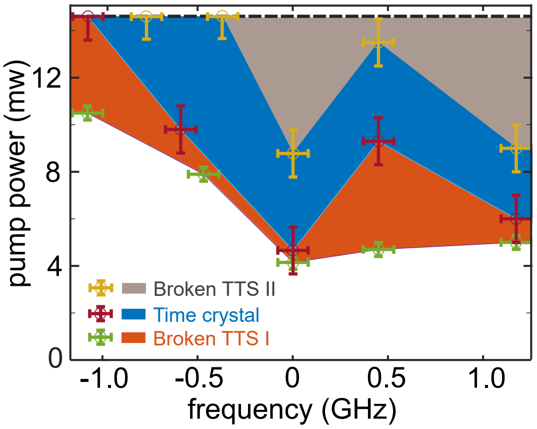

FIG. S5. **Phase diagram of time crystal as a function of laser frequency and pump power.** TTS, time translation symmetry. White area, a phase with stable cw ouput; blue area, the phase of Broken TTS I, where the output becomes dynamically unstable, but without a distinguishable periodic signal; orange area, the phase of time crystal, where a 8.7 kHz periodic signal can be distinguished from the background; gray area, the phase of Broken TTS I, where the output remains dynamically unstable but the 8.7 kHz cycles no longer can be identified due to the increased oscillating background. The cross points are experimentally measured points.

With increasing pump, the $I(t)$ first becomes dynamically unstable, indicating the breaking of continuous time translation symmetry. The spectral range of $I(t)$ is broad with a cut-off frequency ~ 50 MHz [6], which means that $I(t)$ oscillates at the time scale of 10 ns. However, no periodic signal can be identified from the correlation $\langle I(t)I(t+\tau)\rangle$ or the spectra of $I(t)$. In other words, even though the continuous time translation symmetry of our system is broken, there is lack of a temporal order. This regime is named as the phase of broke time translation symmetry I, as shown by the orange area in Fig. S5.

Depending on the f_l, if the $P_{\rm in}$ is further increased, the many-body system can reach a phase with temporal order. For example, for $f_l = 0.00$ GHz, if $P_{\rm in}$ is increased to 5.3 mW, a periodic oscillation of 8.7 KHz can be identified in the already-unstable $I(t)$. For $f_l = 0.50$ GHz and $f_l = 1.12$ GHz, a pump of $P_{\rm in} = 9.3$ mW and 6.0 mW are needed, respectively (dark-red cross points in Fig. S5). Note that under this circumstance, the $I(t)$ is still dynamically unstable and the MHz oscillating components remain there. This regime features the broken time translation symmetry together with a self-generated periodicity, which is named as the phase of time crystal, as shown by the blue area in Fig. S5.

Further increasing $P_{\rm in}$ can lead to the fading of the time crystalline signal. The reason is twofold. On one hand, the 8.7 kHz peak becomes broadened for increasing $P_{\rm in}$ (as shown in Fig. 3a in the manuscript); on the other hand, the oscillation components of $I(t)$ at other frequencies grows with $P_{\rm in}$ (as shown in Note 7, SI). Both the two factors make the periodic signal less distinguishable. Note also that although the time crystalline order fades, the $I(t)$ is still dynamically unstable. This regime is named as the phase of broke time translation symmetry II, as shown by the gray area in Fig. S5.

Note that there are some experimental conditions that limit the determination of the phase transition points. Because the moment when the cw output becomes dynamically unstable, and the moment when the 8.7 kHz can be distinguishable from the background, are lack of a strict standard and rely on experimental experience. Therefore, there are some errors when plotting the phase transition points.

Supplementary Note 7. Phase transitions at different laser frequencies

As shown in the main text that the phase transitions of the inherent time crystal depend on the pump power. For different laser frequency f_l, i.e., placing the laser frequency in different positions of the inhomogeneous absorption line of the Er:Y_2SiO_5, different aspects of such phase-transition processes can be unfolded. Here we show more data of the phase transitions at $f_l = 1.17$ GHz.

When the pump power is low $P_{in} = 5$ mW, there is no oscillating signal in Fig. S6a and S6b. If the pump laser is increased to 6.0 mW, we can see that $I(t)$ becomes dynamically unstable, indicating that the time translation symmetry is spontaneously broken by the erbium dipole-dipole interactions, as shown in Fig. S6c. Its corresponding Fourier spectrum shows a periodic time pattern of 8.7 kHz, as shown in Fig. S6d, indicating the forming of temporal order. Further increasing the pump power to 7.0 mW, the 8.7 kHz peak remains, and the changes in its amplitude and width are not obvious. However, oscillations at other

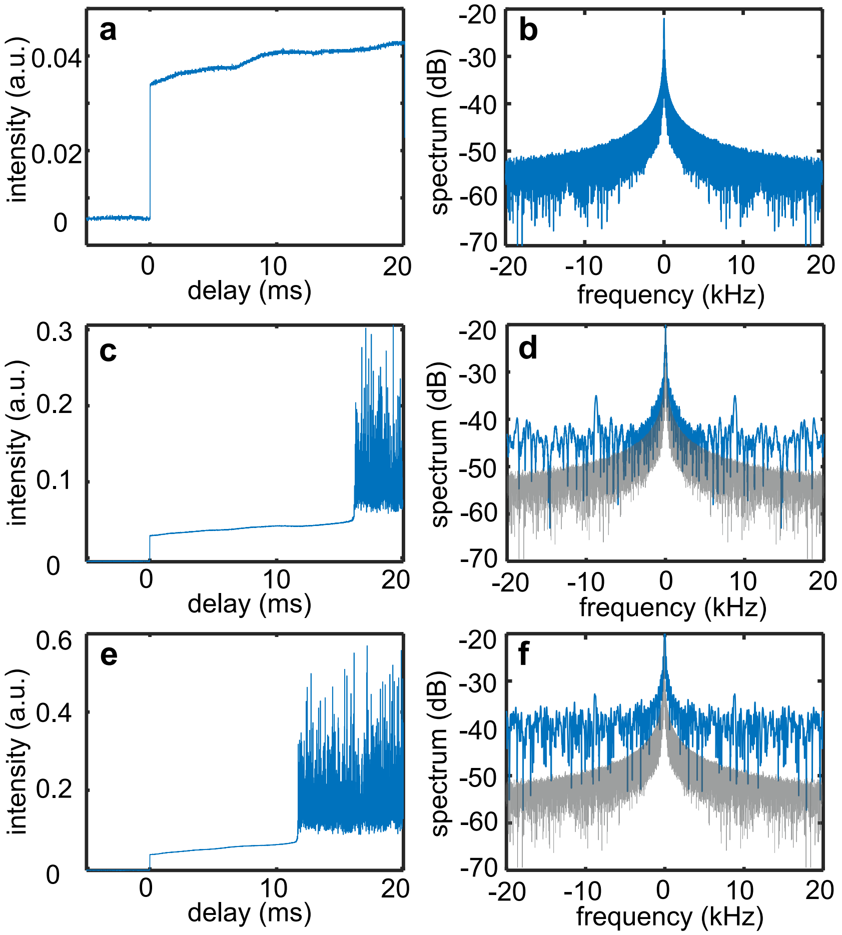

FIG. S6. **Self-organization of time crystal with power dependence.** (a) Time evolution of the measured $I(t)$ for an input of $P_{\mathrm{in}} = 5\,\mathrm{mW}$. (b) The spectrum corresponds to (a). (c) Measured $I(t)$ for $P_{\mathrm{in}} = 6\,\mathrm{mW}$. (d) The spectrum corresponds to (c) with the spectrum of $P_{\mathrm{in}} = 5\,\mathrm{mW}$ in the background. (e) Measured $I(t)$ for $P_{\mathrm{in}} = 7\,\mathrm{mW}$. (f) The spectrum corresponds to (e) with the spectrum of $P_{\mathrm{in}} = 5\,\mathrm{mW}$ in the background. The pump laser frequency is $f_l = 1.17\,\mathrm{GHz}$. The time crystal frequency remains 8.7 kHz.

frequencies become much more significant, as shown in Fig. S6f. Specifically, the spectral intensities at non-zero frequencies (apart from 8.7 kHz) are approximately -45 dB in Fig. S6d and -40 dB in Fig. S6f. The 8.7 kHz peak becomes less obvious as a result of the growing amplitudes of other frequencies. This may finally cause the time crystal to be unidentified. Note that such a transition is different from that of Fig. 3a in the main text, where the broadening of the 8.7 kHz peak at strong P_{in} is caused by the high-order effects rather than growing background noise.

Supplementary Note 8. Intrinsic optical instability without time crystalline order.

The measured $I(t)$ for $P_{\mathrm{in}} = 8\,\mathrm{mW}$ and $f_l = 0.50\,\mathrm{GHz}$ corresponding to Fig. 3a in the manuscript is shown in Fig. S7. After the laser is switched on at $t = 0\,\mathrm{ms}$, the system takes approximately 14 ms to reach a phase of intrinsic optical instability. However, there is no temporal periodicity in $I(t)$ under this circumstance, as evidenced by the lack of peak in its spectrum measured in the long-time limit, which is shown as the orange line in Fig. 3A in the main text.

Supplementary Note 9. Data analysis using cross-correlation function

Here we present the method to obtain the phase information of $I(t)$, as shown in Fig. 3C of the main text. Here we consider a periodic function with both amplitude noise and phase noise such that

$$f(t) = [1 + a(t)] \cdot \cos[\omega_0 t + \phi(t)], \tag{S.29}$$

where ω_0 is the time crystal frequency, $a(t)$ is the amplitude noise, and $\phi(t)$ is the phase noise. The function $f(t)$ is used to

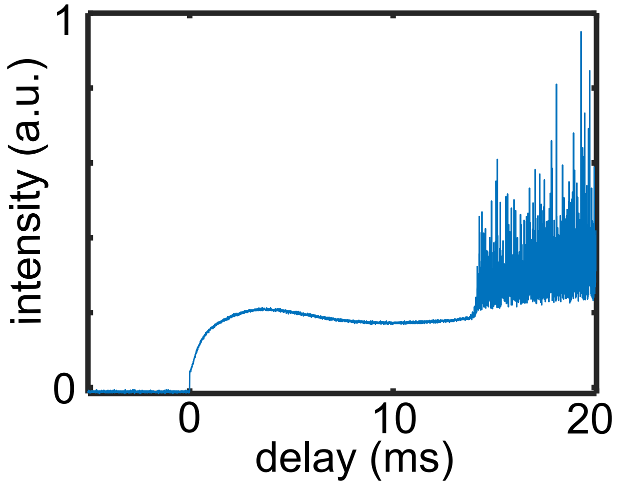

FIG. S7. **Intrinsic optical instability without time crystalline order**. Measured $I(t)$ of $P_{in} = 8\,\mathrm{mW}$ and $f_l = 0.5\,\mathrm{GHz}$. The laser field is switched on at $t = 0\,\mathrm{ms}$.

represent the measured $I(t)$ in experiments. It is worth highlighting here the frequency characteristics of the different parts in $f(t)$:

- The time crystal frequency $\omega_0 = 8.7\,\mathrm{kHz}$, which is obtained from the autocorrelation and the Fourier spectra of $I(t)$, as shown in Fig. 2 in the main text.
- The intrinsic instability $a(t)$ is considered as a rapidly-varying function of time. The typical frequency of $a(t)$ is on the order of tens of MHz, as detailed in the literature[6].
- The phase noise $\phi(t)$ is a slowly-varying function of time. According to the $\sim$ 100 Hz linewidth measured in the Fig. 3c in the main text, the corresponding time scale is on the order of milliseconds.

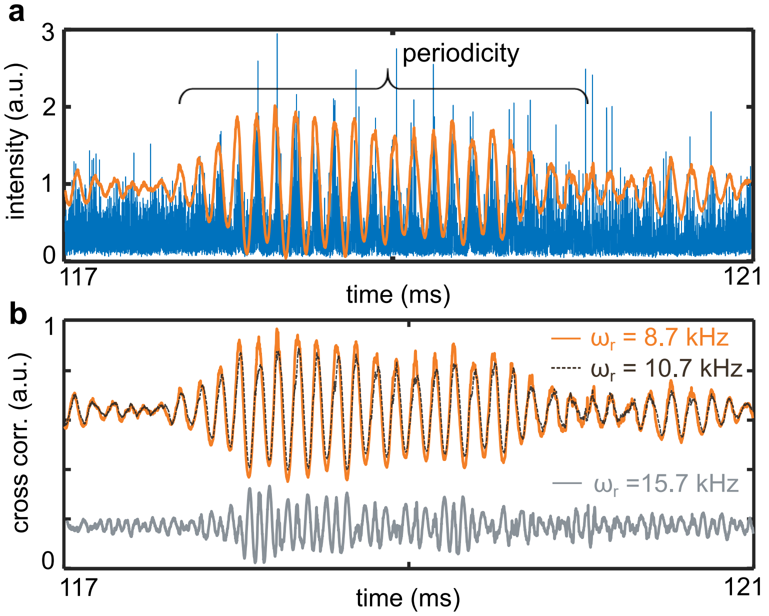

FIG. S8. **Validity of the analysis method.** (a) Comparison of the measured $I(t)$ and the cross-correlation of $F(\tau)$. Blue, the $I(t)$ from 117 ms to 121 ms. Orange, the cross-correlation $F(\tau) = \langle I(t)r(t-\tau)\rangle$ with a delay τ varying from 117 ms to 121 ms. $\omega_r = 8.7\,\mathrm{kHz}$ and the integration time $T = 0.125\,\mathrm{ms}$. (b) Weak dependence of $F(\tau)$ on the reference frequency ω_r. Orange solid, $F(\tau)$ is calculated with a reference frequency of $\omega_r = 8.7\,\mathrm{kHz}$, which is identical to the orange curve in a. Black dashed, $F(\tau)$ calculated with $\omega_r = 10.7\,\mathrm{kHz}$. Gray solid, $F(\tau)$ calculated with $\omega_r = 15.7\,\mathrm{kHz}$. The $\omega_r = 15.7\,\mathrm{kHz}$ curve is vertically shifted for clarity. The amplitudes of the three $F(\tau)$ are in scale.

Based on the different time scales involved in $f(t)$, we use an integration method to extract the period and the phase information hidden in the noisy $f(t)$. We first construct a reference pulse function with a frequency of ω_r:

$$r(t) = \cos(\omega_r t) \cdot \text{rect}(t), \quad \text{with} \quad \text{rect}(t) = \begin{cases} 0, & \text{if} \quad t < 0 \\ 1, & \text{if} \quad 0 \leq t \leq T \\ 0, & \text{if} \quad t > T \end{cases} \tag{S.30}$$

The rect(t) is a rectangle function with an open duration of T, and T represents the integration time of our method. The cross-correlation function between $f(t)$ and $r(t)$ is defined as

$$\begin{aligned} F(\tau) &= \int_{-\infty}^{+\infty} f(t) r(t-\tau) dt \\ &= \int_{\tau}^{\tau+T} [1 + a(t)] \cdot \cos[\omega_0 t + \phi(t)] \cos[(\omega_r (t-\tau)] dt \\ &= \int_{\tau}^{\tau+T} \frac{1 + a(t)}{2} \cos[(\omega_0 + \omega_r)t - \omega_r \tau + \phi(t)] dt \\ &+ \int_{\tau}^{\tau+T} \frac{1 + a(t)}{2} \cos[(\omega_0 - \omega_r)t + \omega_r \tau + \phi(t)] dt. \end{aligned} \tag{S.31}$$

Typically we choose $T \sim 0.1$ ms. As aforementioned, $a(t)$ varies rapidly at frequencies $O(10\,\text{MHz})$. Thus any terms involved the fast oscillating $a(t)$ is averaged to near zero when calculating the integral. The same conclusion also holds for the term with $\cos(\omega_0 + \omega_r)t$. Using these two approximations, we then have

$$F(\tau) \approx \int_{\tau}^{\tau+T} \frac{1}{2} \cos[(\omega_0 - \omega_r)t + \omega_r \tau + \phi(t)] dt. \tag{S.32}$$

Since $\phi(t)$ is a slowly-varying function of time on the order of 1 ms, and the integration time $T \sim 0.1$ ms, we can replace $\phi(t)$ with $\phi(\tau)$ in the above equation such that

$$\begin{aligned} F(\tau) &\approx \int_{\tau}^{\tau+T} \frac{1}{2} \cos[(\omega_0 - \omega_r)t + \omega_r \tau + \phi(\tau)] dt \\ &= \frac{1}{\omega_0 - \omega_r} \sin[(\omega_0 - \omega_r)T/2] \cdot \cos[\omega_0 \tau + \omega_0 T/2 + \phi(\tau)]. \end{aligned} \tag{S.33}$$

At the limit that $(\omega_0 - \omega_r)T \to 0$, we obtain that

$$F(\tau) \approx \frac{T}{2} \cdot \cos[\omega_0 \tau + \omega_0 T/2 + \phi(\tau)] \tag{S.34}$$

Neglecting a constant phase of $\omega_0 T/2$, we finally have

$$F(\tau) \propto \cos[\omega_0 \tau + \phi(\tau)] \tag{S.35}$$

Comparing Eq (S.35) and Eq (S.29), we can reproduce the period and the phase information of $f(t)$ by choosing different τ (with a time resolution determined by T). To confirm the method's validity, we calculate the cross-correlation between the $I(t)$ from 117 ms to 121 ms in Fig. S8a and a reference function with $\omega_r = 8.7$ kHz and $T = 0.125$ ms. The result is plotted as an orange line on top of Fig. S8a. It is obvious that the cross-correlation $F(\tau)$ reproduces the period and the phase information of $I(t)$ from a background of high frequency noise.

Note also that the analysis method here is not sensitive to the reference frequency ω_r. This property can be seen from Eq. (S.34), which does not depend on ω_r. That is to say, one does not have to precisely pre-measure ω_0 and set $\omega_r = \omega_0$ during the data analysis. As long as $(\omega_r - \omega_0)T \to 0$, Eq. (S.34) holds. We present $F(\tau)$ of three different ω_r in Fig. S8b. While the result of $\omega_r = 8.7$ kHz indicates a periodic oscillation of $I(t)$, the period and the phase information of $I(t)$ can also be revealed, almost identically, by the result of $\omega_r = 10.7$ kHz. However, if ω_r is further increased to 15.7 kHz, the $F(\tau)$ becomes improper and is no longer useful in extracting the phase information, as shown by the gray curve in Fig. S8b.

Supplementary Note 10. Periodicity at different delays

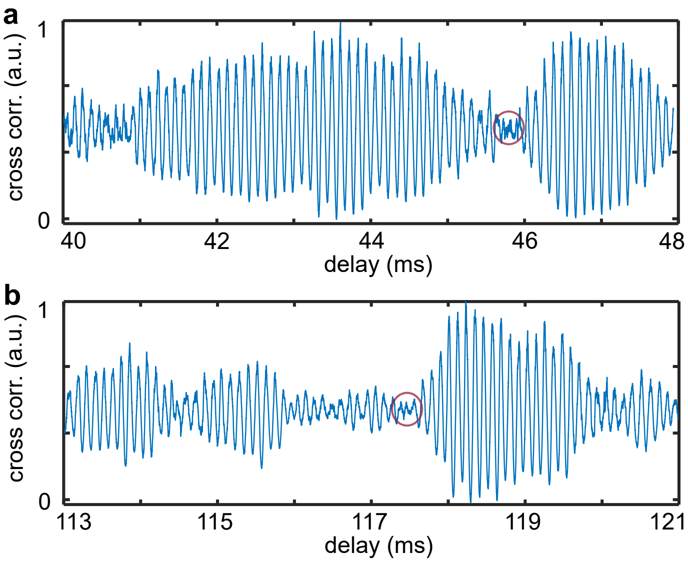

FIG. S9. **The correlation function $F(\tau)$ at different delay times.** (a) $F(\tau)$ of delay τ from 40-48 ms. A time point with phase discontinuity can be identified at approximately 46 ms. (b) $F(\tau)$ of delay τ from 113-121 ms. A time point with phase discontinuity can be identified at approximately 117.5 ms.

Using $F(\tau)$ detailed in Note 8, one can reveal the periodicity and the phase information of $I(I)$ more clearly than the raw data $I(t)$ itself. Exemplified in Fig. S9 are the cross correlation $F(\tau)$ for different delay times of the same $I(t)$ in Fig. 3C in the manuscript. It is clear that oscillations with a frequency of 8.7 kHz consecutively arise from 40 ms to 121 ms, as shown in Fig. S9a, S9b and Fig. 3C in the manuscript. Instead of being a constant in the long time scale, the phase of oscillations is a slowly-varying function of time, showing phase discontinuities at random time positions, as marked by the red circles in Fig. S9a and S9b. As discussed in the manuscript, such phase discontinuities result from the phase noise of our fiber laser.

The phase change consistently occurs when the 8.7 kHz signal slowly envelopes to a small amplitude (it never occurs when the 8.7 kHz oscillations are of large amplitude), indicating a continuous phase change rather than a sudden jump. However, characterizing the lasers phase noise at 100 Hz scales is challenging, and we lack the necessary equipment for a conclusive determination of its nature.

Supplementary Note 11. Phase discontinuities in the theoretical model

To understand the effect of the phase noise in driving to the inherent time crystal, we have calculated the response of $\rho(t)$ for a laser pump with phase shift at some specific moments. The results are shown in Fig. S10. We can see that the system takes approximately 4 ms to self-organize to temporal order, as shown by the left inset of Fig. S10. At t = 5.6 ms and t = 11.2 ms, phase shifts of π are imposed on the optical driving field, as marked by the red dashed lines in Fig. S10. These phase perturbations break the balance of the system. The $\rho(t)$ thus losses its temporal order and takes approximately 4 ms to self-organize to another one, as shown by the right inset of of Fig. S10. The period of this new temporal order is the same as the previous one. Still, there is a phase discontinuity between them. The calculated results of imposed phase discontinuities in the driving field agree well with our experiment: although the oscillating $I(t)$ persists, the phase noise of the laser breaks $I(t)$ into segments on the order of milliseconds.

Supplementary Note 11. Differences between self pulsing and time crystal

It is well-known that self-pulsing effect can occur in erbium-doped fiber lasers [17–21], in which the laser output power fluctuates in a periodic or quasi-periodic manner without any external modulation. The fluctuations in the output power are generally caused by the competition between the gain of the laser medium and the losses due to the cavity's various components. As a result, the net gain inside the cavity is periodically modulated and generate fluctuations in the laser output. Although our time crystal and self pulsing both exhibit periodic modulations in their outputs, they are fundamentally different and have different underlying physical mechanisms. The most important differences are:

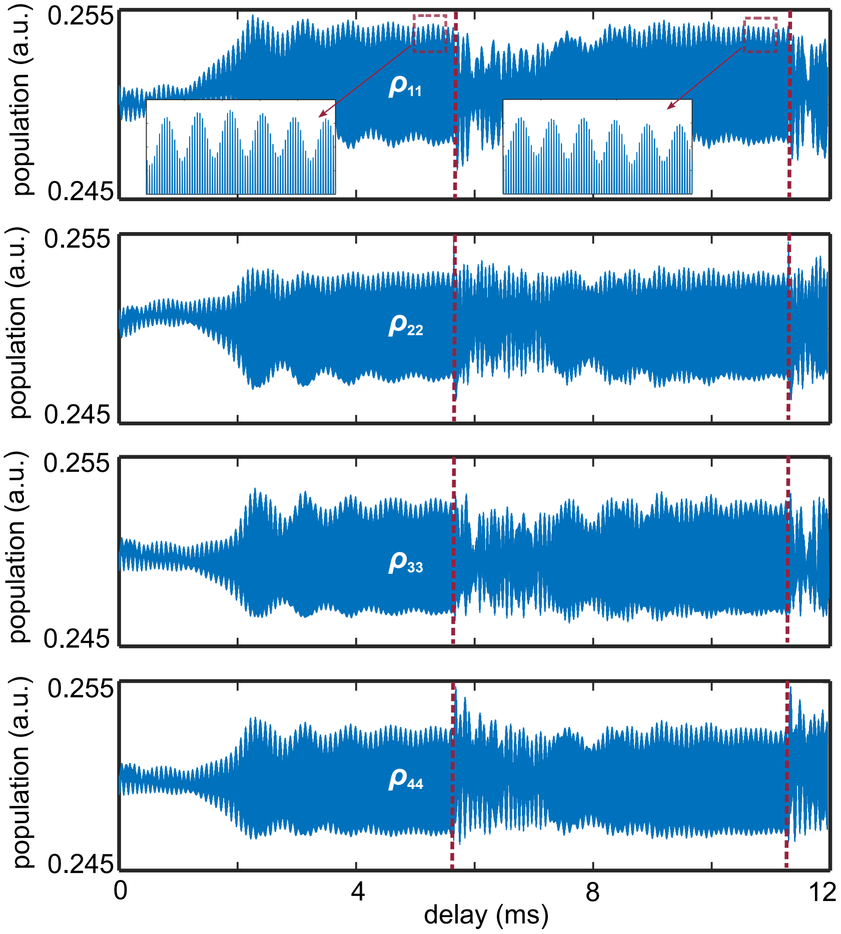

FIG. S10. **Calculation of $\rho_{nn}(t)$ with phase noise in the driving field.** The dynamic behaviors of the populations in the four levels as noted. $\rho_{nn}(t)$ shows persistent oscillations in the long time limit. It takes approximately 4 ms to form an inherent time crystal phase. At $t = 5.6$ ms and $t = 11.2$ ms, as marked by the red dashed line, phase shifts of π are imposed to the optical driving field.

a. The experimental setups are different. Optical cavity is a necessary for the well-know self-pulsing effects in erbium-doped fibre lasers. Our experimental setup differs significantly. One end of our sample is antireflection coated with a reflectivity of less than 0.8%, preventing the formation of a similar cavity. In addition, our sample is of 12 mm long. In contrast, the fibre laser systems that manifest self pulsing effect typically have a length on the order of meters, that is, two-orders-of-magnitude difference. Thus, the achievable optical gain in our crystal, given that the erbium concentrations are similar, is much less than that in fibre laser systems due to these factors. These differences distinguish our experimental setup from traditional fiber laser systems and should be taken into account in any comparisons or analyses.

b. The experimental pumping pumping conditions different. Self-pulsing behaviour in 1.5 μm erbium-doped fiber lasers are observed with pumping at shorter wavelength, such as 514 nm, 810 nm or 980 nm. The 1.5 μm erbium laser which are self-pulsing when pumped at 980 nm becomes stable when pumped at 1490 nm or longer. In fact, it has been demonstrated that by adding an auxiliary 1.5 μm pump with only 3% of the lasing power, the self-pulsation in the system can be significantly suppressed [18, 19]. Our results were observed with erbium ions pumped by 1.5 μm laser, which can effectively eliminate the self-pulsing effect.

c. The experimental phenomena are different. In self-pulsing experiments, increasing the pumping power can drive the laser output from a stable to a periodically modulated regime, and further increase can lead to chaotic behavior. However, in our experiments, increasing the pump power first results in dynamically unstable output with frequency response up to ~50 MHz [6]. With additional pump power, a periodic oscillation of 8.7,kHz emerges. Beyond that, higher pump power brings the system to another unstable phase. Moreover, the 8.7 kHz in our experiment is unaffected by the increase of the pump power. This observation is in contrast to the case of self pulsing, whose frequency typically depends on the pump power. As a result, the behavior of our system under increased pumping power differs significantly from that of

self-pulsing systems (see Supplementary Note 6, SI).

d. The physical origins are different. The self-pulsing effect is typically the result of the dymamic interplay between the optical gain and the absorption or losses in laser systems. Under these circumstances, the net optical gain can be temporally modulated, leading to the switching on-and-off of the laser output. However, our experiment does not involve a cavity or optical net gain, and cannot be explained as the switching on-and-off of the cavity output. Additionally, the up to ~50 MHz frequency response observed in our time crystal phase exceeds the energy transfer rate between the erbium ions in the self-pulsing model. Therefore, the temporal order observed in our experiments likely arises from another mechanism instead of the well-known self-pulsing effect.

---

[1] S. Diehl, A. Tomadin, A. Micheli, R. Fazio, and P. Zoller, Physical Review Letters **105**, 015702 (2010).
[2] T. E. Lee, H. Häffner, and M. C. Cross, Physical Review A **84**, 031402 (2011).
[3] A. Karabanov, D. C. Rose, W. Köckenberger, J. P. Garrahan, and I. Lesanovsky, Physical Review Letters **119**, 150402 (2017), arXiv:1703.07159.
[4] C. Carr, R. Ritter, C. G. Wade, C. S. Adams, and K. J. Weatherill, Physical Review Letters **111**, 113901 (2013), arXiv:1302.6621.
[5] R. L. Ahlefeldt, D. L. McAuslan, J. J. Longdell, N. B. Manson, and M. J. Sellars, Physical Review Letters **111**, 240501 (2013).
[6] Y.-H. Chen, S. P. Horvath, J. J. Longdell, and X. Zhang, Physical Review Letters **126**, 110601 (2021), arXiv:1910.14298.
[7] J. Huang, J. M. Zhang, A. Lezama, and T. W. Mossberg, Physical Review Letters **63**, 78 (1989).
[8] N. Ohlsson, R. K. Mohan, and S. Kroll, Optics Communications **201**, 71 (2002).
[9] J. J. Longdell, M. J. Sellars, and N. B. Manson, Physical Review Letters **93**, 130503 (2004).
[10] M. P. Hehlen, H. U. Güdel, Q. Shu, J. Rai, S. Rai, and S. C. Rand, Physical Review Letters **73**, 1103 (1994).
[11] R. A. Vlasov, A. M. Lemeza, and M. G. Gladush, Laser Physics Letters **10**, 045401 (2013).
[12] P. Kongkhambut, J. Skulte, L. Mathey, J. G. Cosme, A. Hemmerich, and H. Keßler, Science **377**, 670 (2022), arXiv:2202.06980.
[13] B. Buča, J. Tindall, and D. Jaksch, Nature Communications **10**, 1730 (2019), arXiv:1804.06744.
[14] Y. Sun, C. Thiel, R. Cone, R. Equall, and R. Hutcheson, Journal of Luminescence **98**, 281 (2002).
[15] M. Zhong, M. P. Hedges, R. L. Ahlefeldt, J. G. Bartholomew, S. E. Beavan, S. M. Wittig, J. J. Longdell, and M. J. Sellars, Nature **517**, 177 (2015), arXiv:1411.6758.
[16] P. Kongkhambut, J. Skulte, L. Mathey, J. G. Cosme, A. Hemmerich, and H. Keßler, Science **377**, 670 (2022).
[17] F. Sanchez and G. Stephan, Physical Review E **53**, 2110 (1996).
[18] W. H. Loh, Optics Letters **21**, 734 (1996).
[19] L. Luo and P. L. Chu, Optics Letters **22**, 1174 (1997).
[20] R. Rangel-Rojo and M. Mohebi, Optics Communications **137**, 98 (1997).
[21] A. F. El-Sherif and T. A. King, Optics Communications **208**, 381 (2002).
